# Supplementary material for: Mediterranean diet assessment challenges: Validation of the Croatian Version of the 14-item Mediterranean Diet Serving Score (MDSS) Questionnaire
Source: PLoS One. 2021 Mar 1;16(3):e0247269. doi: 10.1371/journal.pone.0247269 (PMC7920370; doi:10.1371/journal.pone.0247269)
Supplement: S1 Checklist — (DOCX) [file pone.0247269.s001.docx]

STROBE Statement—checklist of items that should be included in reports of observational studies

|  | | Item No. | | Recommendation | Page  No. | | Relevant text from manuscript |
| --- | --- | --- | --- | --- | --- | --- | --- |
| **Title and abstract** | | 1 | | (*a*) Indicate the study’s design with a commonly used term in the title or the abstract | 2 | | The aim of this cross-sectional study was to assess reliability and validity of the Croatian version of the 14-item Mediterranean Diet Serving Score (MDSS), using the Mediterranean Diet Adherence Screener (MEDAS) as the comparator. |
|  |  |  |  | (*b*) Provide in the abstract an informative and balanced summary of what was done and what was found | 2 | | Test-retest reliability and validity of the MDSS score were tested using intra-class correlation coefficients (ICC), while Cohen’s kappa statistic was used to test correct classification of subjects into MD adherent/non-adherent category. A very good reliability was shown for the overall MDSS score (ICC=0.881 [95% CI 0.843-0.909]), and a moderate reliability for the binary adherence (κ=0.584). Concurrent validity of the MDSS was also better when expressed as a total score (ICC=0.544 [0.439-0.629]) as opposed to the adherence (κ=0.223), with similar result in the confirmatory sample (ICC=0.510 [0.384-0.610]; κ=0.216). |
| Introduction | | | | | | |  |
| Background/rationale | | 2 | | Explain the scientific background and rationale for the investigation being reported | 3-5 | | Even though Croatia is one of the seven Mediterranean countries that participated in the process of inscription of the MD to the UNESCO’s representative list of the intangible cultural heritage of humanity, no study has so far tested any dietary questionnaire regarding its’ validity for assessing the MD adherence in adults in Croatia. Only one previous study has assessed the reliability of the KIDMED questionnaire, but it was done in a sample of students from the continental part of Croatia, and not from the Mediterranean region [[39](#_ENREF_39)]. |
| Objectives | | 3 | | State specific objectives, including any prespecified hypotheses | 5 | | This study aims to evaluate validity (accuracy) and reliability of the Croatian version of the short, 14-item Mediterranean Diet Serving Score (MDSS) questionnaire, compared to the Mediterranean Diet Adherence Screener (MEDAS), based on a sample of students from the University of Split, Croatia. |
| Methods | | | | | | |  |
| Study design | | 4 | | Present key elements of study design early in the paper | 6 | | This cross-sectional study was carried out in Split, Croatia, the largest city on the coast of the Adriatic Sea. |
| Setting | | 5 | | Describe the setting, locations, and relevant dates, including periods of recruitment, exposure, follow-up, and data collection | 6 | | We included two samples in order to assess psychometric properties of the Croatian version of a short MD questionnaire. The initial, exploratory sample of medical students from the University of Split School of Medicine was used for reliability testing (test-retest repeatability) and concurrent and construct validity of the Croatian version of the short MDSS questionnaire. A total of 377 medical students enrolled in the first, third and fifth study year (out of six study years) were sampled during the period of December 2018 - October 2019, with the overall response rate of 80.2%. The second, independent sample was used to confirm the initial MDSS questionnaire validity results and to investigate its’ predictive validity. This independent confirmatory sample consisted of 320 students from the University Department of Health Studies (nurses, lab technicians, radiology technicians, physiotherapists; response rate 81.2%) sampled during the period of May - December 2019. |
| Participants | | 6 | | (*a*) *Cohort study*—Give the eligibility criteria, and the sources and methods of selection of participants. Describe methods of follow-up  *Case-control study*—Give the eligibility criteria, and the sources and methods of case ascertainment and control selection. Give the rationale for the choice of cases and controls  *Cross-sectional study*—Give the eligibility criteria, and the sources and methods of selection of participants | 6 | | Inclusion criteria were age over 18 years, both genders, and the willingness to provide the informed consent. There were no exclusion criteria. … The students were invited to participate in the study during their mandatory courses, in order to ensure the highest possible response rate. After the initial explanation of the purpose and procedures of the study, students who decided to participate were asked to sign the informed consent. Medical students were tested at two time points and Health studies students were tested just once, but using a more detailed survey. |
|  |  |  |  | (*b*) *Cohort study*—For matched studies, give matching criteria and number of exposed and unexposed  *Case-control study*—For matched studies, give matching criteria and the number of controls per case | / | |  |
| Variables | | 7 | | Clearly define all outcomes, exposures, predictors, potential confounders, and effect modifiers. Give diagnostic criteria, if applicable | 9-10 | | Scoring criteria for both questionnaires are presented in detail in Table 1, and they were done according to originally proposed criteria |
| Data sources/ measurement | | 8* | | For each variable of interest, give sources of data and details of methods of assessment (measurement). Describe comparability of assessment methods if there is more than one group | 8-11 | | All of the data were obtained via questionnaire, described in Methods section, while both MD questionnaires are presented in Supplementary Table 1 |
| Bias | | 9 | | Describe any efforts to address potential sources of bias | 6 | | High response rate, use of two samples: initial and confirmatory sample (“The second sample was used to replicate results and confirm the initial MDSS questionnaire validity results and to investigate its’ predictive validity.”) |
| Study size | | 10 | | Explain how the study size was arrived at | 13 | | Due to the missing data from the MD questionnaires, we excluded 17 medical students from the initial exploratory sample (see Fig 2). This resulted in the sample size of 360 students at the first time point, included in concurrent validity analysis, while 210 of these students were available during the second time point (retest), and they were included in the test-retest reliability analysis. This was appropriate sample size, based on the estimate of an ideal subject to questionnaire item ratio being between 10:1 and 20:1. |
| Continued on next page Quantitative variables | 11 | | Explain how quantitative variables were handled in the analyses. If applicable, describe which groupings were chosen and why | | 11 | According to the original study, people with a score of ≥13.5 on the MDSS scale can be considered as adherent to the principles of the MD, which we rounded up to 14 points (Table 1). … There are two ways to categorize the overall MEDAS score. Subjects can be divided into 3 subgroups, where the score of ≤5 points indicates low adherence, 6–9 indicates moderate adherence and ≥10 points indicates high level of adherence to the principles of the MD (Table 1) [[25](#_ENREF_25), [29](#_ENREF_29)]. Additionally, a cut-off score of ≥8 points has been used to denote adherence to the principles of the MD, while MEDAS score of ≤7 points represents MD non-adherence. | |
| Statistical methods | 12 | | (*a*) Describe all statistical methods, including those used to control for confounding | | 12-13 | Categorical variables were presented as absolute numbers and percentages. Numerical variables were mostly non-normally distributed (tested by Kolmogorov–Smirnov test), and they were presented as medians and interquartile ranges (IQR). Differences between groups were tested using chi-square test for categorical variables, and Mann–Whitney U test for numerical variables. Spearman rank test was used to test bivariate correlation between numerical variables.  Test-retest reliability was tested using intra-class correlation coefficients (ICC; two-way mixed model) and Spearman rank test for both MDSS and MEDAS overall scores. Based on the ICC estimates, values <0.50 were considered to show poor agreement, values between 0.50 and 0.75 as moderate, between 0.75 and 0.90 as good agreement, while values greater than 0.90 were regarded as excellent reliability [[44](#_ENREF_44)]. Cohen’s kappa statistic was used for assessing agreement between test-retest classification of subjects into tertiles and for MD adherence/non-adherence classification based on the appropriate cut-off points available in Table 1. According to McHugh et al., values ≤0 indicate that there is no agreement, and values 0.01–0.20 indicate that there is a slight agreement, 0.21–0.40 as fair agreement, 0.41– 0.60 as moderate agreement, 0.61–0.80 as substantial agreement, and 0.81–1.00 as almost perfect agreement [[45](#_ENREF_45)]. Additionally, test-retest agreement was calculated using kappa statistic for all of the separate food groups [[45](#_ENREF_45)].  Concurrent validity of MDSS index was also tested using ICC, Spearman rank test and Cohen’s kappa statistic, against MEDAS index, both for the first testing time, and for the retest. Despite methodological limitations, Spearman’s rank test was calculated to provide comparison with previous studies.  We also applied the Principal Component Analysis (PCA) to both MD questionnaires to test construct validity and to identify food groups (factors), using Varimax rotation and the cut-off of >0.30 for absolute factor loadings to suppress small coefficients. The suitability of the data for factor analysis was tested by the Kaiser–Meyer–Olkin measure of sampling adequacy (≥0.60) and Bartlett’s test of sphericity (P<0.05). Factors with an Eigenvalue ≥1.0 were retained, and total explained variance was recorded. | |
|  |  |  | (*b*) Describe any methods used to examine subgroups and interactions | | 12-13 | Cohen’s kappa statistic was used for assessing agreement between test-retest classification of subjects into tertiles and for MD adherence/non-adherence classification based on the appropriate cut-off points available in Table 1. | |
|  |  |  | (*c*) Explain how missing data were addressed | | 13 | Due to the missing data from the MD questionnaires, we excluded 17 medical students from the initial sample, and 21 students from the confirmatory sample (see Fig 2). | |
|  |  |  | (*d*) *Cohort study*—If applicable, explain how loss to follow-up was addressed  *Case-control study*—If applicable, explain how matching of cases and controls was addressed  *Cross-sectional study*—If applicable, describe analytical methods taking account of sampling strategy | | 12-13 |  | |
|  |  |  | (*e*) Describe any sensitivity analyses | | 9 | Additionally, we used the Warwick–Edinburgh Mental Well-being Scale (WEMWBS), a validated questionnaire used for measuring mental well-being, especially focusing on the positive aspects of mental health [[43](#_ENREF_43)]. This questionnaire was translated to Croatian using the same ISPOR procedure [[40](#_ENREF_40)], and it was applied in both exploratory and confirmatory sample. The purpose of WEMWBS questionnaire was to serve as a non-dietary reliability comparator and as an outcome in the predictive validity analysis in order to investigate the association between the MD and well-being in students. | |
| Results | | | | | | | |
| Participants | 13* | | (a) Report numbers of individuals at each stage of study—eg numbers potentially eligible, examined for eligibility, confirmed eligible, included in the study, completing follow-up, and analysed | | 7 | Figure 2 | |
|  |  |  | (b) Give reasons for non-participation at each stage | | 7 | Figure 2 | |
|  |  |  | (c) Consider use of a flow diagram | | 7 | Figure 2 | |
| Descriptive data | 14* | | (a) Give characteristics of study participants (eg demographic, clinical, social) and information on exposures and potential confounders | | 14-16 | Table 2 | |
|  |  |  | (b) Indicate number of participants with missing data for each variable of interest | | 14 | The study included 248 women (71%) and 102 men (29%), while 10 students didn’t provide information on the gender. | |
|  |  |  | (c) *Cohort study*—Summarise follow-up time (eg, average and total amount) | | / |  | |
| Outcome data | 15* | | *Cohort study*—Report numbers of outcome events or summary measures over time | | */* |  | |
|  |  |  | *Case-control study—*Report numbers in each exposure category, or summary measures of exposure | | */* |  | |
|  |  |  | *Cross-sectional study—*Report numbers of outcome events or summary measures | | 14, 15, 20 | Table 2, Table 7 | |
| Main results | 16 | | (*a*) Give unadjusted estimates and, if applicable, confounder-adjusted estimates and their precision (eg, 95% confidence interval). Make clear which confounders were adjusted for and why they were included | | Not applicable |  | |
|  |  |  | (*b*) Report category boundaries when continuous variables were categorized | | 10, 11 | Table 1 | |
|  |  |  | (*c*) If relevant, consider translating estimates of relative risk into absolute risk for a meaningful time period | | Not applicable |  | |

Continued on next page

| Other analyses | 17 | Report other analyses done—eg analyses of subgroups and interactions, and sensitivity analyses | - |  |
| --- | --- | --- | --- | --- |
| Discussion | | | | |
| Key results | 18 | Summarise key results with reference to study objectives | 23 | We showed a very good reliability of the overall MDSS score, while the reliability of the MD adherence as a binary variable was moderate. Validity of the MDSS index, compared to the MEDAS index as a referent point, was also better when expressed as a total score than adherence. These results were replicated in our confirmatory sample, verifying our findings that the MDSS questionnaire is a reasonably valid instrument for the MD assessment in Croatia. |
| Limitations | 19 | Discuss limitations of the study, taking into account sources of potential bias or imprecision. Discuss both direction and magnitude of any potential bias | 26-27 | One of the limitations of this study is a cross-sectional design for the part of the study investigating predictive validity of the MD questionnaires. Furthermore, data collection was carried out in such a way that subjects were required to recall their eating habits, which could have resulted in the recall bias. Most importantly, we didn’t use face to face interviews like previous validation studies did [[29](#_ENREF_29), [37](#_ENREF_37)], but instead we used a self-administered questionnaire. We believed that our anonymous and self-administering approach would enhance the response rate and facilitate honest responses from students, while not substantially diminishing credibility and reliability of the data (a facilitator was always present and students could inquire about any uncertainties).  It is also important to note that our sample included a younger population comprised exclusively of students, who are mostly healthy, while previous validation studies were mostly carried out on a sample of older adults at risk for various chronic diseases[[29](#_ENREF_29), [32](#_ENREF_32), [34](#_ENREF_34)]. |
| Interpretation | 20 | Give a cautious overall interpretation of results considering objectives, limitations, multiplicity of analyses, results from similar studies, and other relevant evidence | 27 | We demonstrated that a short version of the MDSS questionnaire is highly reliable and reasonably valid instrument for the assessment of the adherence to the overall Mediterranean dietary pattern in Croatia. It demonstrated the best performance of MDSS when used as a numeric score, even in the population with low MD adherence. Despite above-mentioned limitations, Croatian version of the short 14-items MDSS questionnaire can be used for rapid assessment of adherence to the MD in Croatia, possibly both in research and in clinical practice. |
| Generalisability | 21 | Discuss the generalisability (external validity) of the study results | 27 | The strengths of the study include a strict methodological framework, two relatively large and independent samples with high response rate (≥80%) from Dalmatia county, which were assumed to have a more uniform and traditional eating habits. This is the first study to compare the MDSS with MEDAS questionnaire for validation purposes and the first validation study of the Croatian version of the short MD questionnaire for adult population. |
| Other information | |  | | |
| Funding | 22 | Give the source of funding and the role of the funders for the present study and, if applicable, for the original study on which the present article is based | There was no funding | The study received no funding; it was performed within the science popularization project awarded to IK by the Ministry of Science and Education of the Republic of Croatia. |

*Give information separately for cases and controls in case-control studies and, if applicable, for exposed and unexposed groups in cohort and cross-sectional studies.

**Note:** An Explanation and Elaboration article discusses each checklist item and gives methodological background and published examples of transparent reporting. The STROBE checklist is best used in conjunction with this article (freely available on the Web sites of PLoS Medicine at http://www.plosmedicine.org/, Annals of Internal Medicine at http://www.annals.org/, and Epidemiology at http://www.epidem.com/). Information on the STROBE Initiative is available at www.strobe-statement.org.
