# Supplementary material for: Mediterranean diet assessment challenges: Validation of the Croatian Version of the 14-item Mediterranean Diet Serving Score (MDSS) Questionnaire
Source: PLoS One. 2021 Mar 1;16(3):e0247269. doi: 10.1371/journal.pone.0247269 (PMC7920370; doi:10.1371/journal.pone.0247269)
Supplement: S1 Table — (DOCX) [file pone.0247269.s002.docx]

**S1 Table. Croatian and English versions of the MDSS [26] and MEDAS [23] questionnaires**

**KRATKI MDSS UPITNIK** (Croatian version [26])

Molimo Vas, zaokružite broj **za svaku** od navedenih skupina namirnica, ovisno o tome koliko često ste ju uobičajeno upotrebljavali tijekom posljednje godine dana.

| **NAMIRNICE** | **svaki dan, 2 ili više puta dnevno** | **svaki dan, 1 dnevno** | **3 puta tjedno** | **2 puta tjedno** | **1 tjedno** | **1 mjesečno** | **rijetko ili nikada** |
| --- | --- | --- | --- | --- | --- | --- | --- |
| Žitarice, sve vrste (kruh, tjestenina, riža, ječam, zob, kukuruz, heljda, pahuljice, i drugo …) | 1 | 2 | 3 | 4 | 5 | 6 | 7 |
| Krumpir | 1 | 2 | 3 | 4 | 5 | 6 | 7 |
| Maslinovo ulje (uključujući i pripremu hrane) | 1 | 2 | 3 | 4 | 5 | 6 | 7 |
| Orašasti plodovi (npr. badem, orah, …) | 1 | 2 | 3 | 4 | 5 | 6 | 7 |
| Voće (isključujući voćne sokove) | 1 | 2 | 3 | 4 | 5 | 6 | 7 |
| Povrće | 1 | 2 | 3 | 4 | 5 | 6 | 7 |
| Mlijeko i mliječni proizvodi | 1 | 2 | 3 | 4 | 5 | 6 | 7 |
| Mahunarke (leća, bob, slanutak, grašak, grah i sl.) | 1 | 2 | 3 | 4 | 5 | 6 | 7 |
| Jaja | 1 | 2 | 3 | 4 | 5 | 6 | 7 |
| Riba (i bijela i plava) | 1 | 2 | 3 | 4 | 5 | 6 | 7 |
| Bijelo meso (piletina i puretina) | 1 | 2 | 3 | 4 | 5 | 6 | 7 |
| Crveno meso (svinjetina, teletina, govedina) | 1 | 2 | 3 | 4 | 5 | 6 | 7 |
| Slastice (kolači, slatka peciva, bomboni, čokolada i ostali slatkiši) | 1 | 2 | 3 | 4 | 5 | 6 | 7 |
| Sokovi (i gazirani i negazirani, prirodni), uključujući i vodu s okusom | 1 | 2 | 3 | 4 | 5 | 6 | 7 |
| Vino (u količini: 1 čaša za žene, 1-2 čaše za muškarce) | 1 | 2 | 3 | 4 | 5 | 6 | 7 |

**Short MDSS questionnaire** (English version [26])

Please circle the number **for each** of the listed food groups, depending on how often you have usually consumed it during the last year.

| **FOODS** | **each day, twice or more a day** | **each day, once a day** | **3 times a week** | **2 times a week** | **once a week** | **once a month** | **rarely or never** |
| --- | --- | --- | --- | --- | --- | --- | --- |
| Cereals, all types (bread, pasta, rice, barley, oats, corn, buckwheat, breakfast cereals and other …) | 1 | 2 | 3 | 4 | 5 | 6 | 7 |
| Potato | 1 | 2 | 3 | 4 | 5 | 6 | 7 |
| Olive oil (including for food preparation) | 1 | 2 | 3 | 4 | 5 | 6 | 7 |
| Nuts (e.g. almond, walnut...) | 1 | 2 | 3 | 4 | 5 | 6 | 7 |
| Fresh fruit (excluding fruit juices) | 1 | 2 | 3 | 4 | 5 | 6 | 7 |
| Vegetables | 1 | 2 | 3 | 4 | 5 | 6 | 7 |
| Milk and dairy products | 1 | 2 | 3 | 4 | 5 | 6 | 7 |
| Legumes (lentils, broad beans, chickpeas, peas, beans, etc.) | 1 | 2 | 3 | 4 | 5 | 6 | 7 |
| Eggs | 1 | 2 | 3 | 4 | 5 | 6 | 7 |
| Fish (both white and blue) | 1 | 2 | 3 | 4 | 5 | 6 | 7 |
| White meat (chicken and turkey) | 1 | 2 | 3 | 4 | 5 | 6 | 7 |
| Red meat (pork, veal, beef) | 1 | 2 | 3 | 4 | 5 | 6 | 7 |
| Sweets (cakes, pastry, chocolate and other sweets) | 1 | 2 | 3 | 4 | 5 | 6 | 7 |
| Juices (both carbonated and non-carbonated, natural), including flavored waters | 1 | 2 | 3 | 4 | 5 | 6 | 7 |
| Wine (in quantity: 1 glass for women, 1-2 glasses for men) | 1 | 2 | 3 | 4 | 5 | 6 | 7 |

**MEDAS UPITNIK** (Croatian version [23])

| 1. | Koristite li maslinovo ulje kao glavni izvor masnoća u prehrani?  1) Ne 2) Da |
| --- | --- |
| 2. | Koliko maslinovog ulja konzumirate u određenom danu (uključujući ulje koje se koristi za prženje, za salate, jela izvan kuće, itd.)? __________ velikih žlica |
| 3. | Koliko obroka (porcija) povrća konzumirate dnevno? (1 porcija = 200 g, prilog se smatra kao ½ porcije) _______, od kojih dnevno pojedem ________ porcija sirovog povrća ili u obliku salate |
| 4. | Koliko voća (komada ili šalica u slučaju sitnijeg bobićastog voća, uključujući prirodne voćne sokove) pojedete dnevno? __________ |
| 5. | Koliko porcija crvenog mesa, hamburgera ili mesnih proizvoda (šunka, kobasica, itd.) pojedete dnevno?  (1 porcija = 100-150 g) __________ |
| 6. | Koliko porcija maslaca, margarina ili vrhnja pojedete dnevno?  (1 porcija = 12 g) __________ |
| 7. | Koliko slatkih ili gaziranih pića dnevno pijete? __________ |
| 8. | Koliko vina pijete tjedno? __________ čaše |
| 9. | Koliko porcija mahunarki (grah, grašak, slanutak…) pojedete tjedno?  (1 porcija = 150 g) __________ |
| 10. | Koliko porcija ribe ili školjki pojedete tjedno?  (1 porcija = 100-150 g ribe; 4-5 komada ili 200 g školjaka) ____________________________ |
| 11. | Koliko puta tjedno konzumirate slatkiše ili kolače (ne domaće), kao što su slatka peciva, kolači, keksi? __________ |
| 12. | Koliko porcija orašastih plodova (uključujući kikiriki) pojedete tjedno?  (1 porcija = 30 g) ________________ |
| 13. | Jedete li radije piletinu, puretinu ili kunića, umjesto teletine, svinjetine, hamburgera ili kobasica?  1) Ne 2) Da |
| 14. | Koliko puta tjedno jedete povrće, tjesteninu, rižu ili druga jela pripremljena s umakom od povrća od rajčice, luka, češnjaka ili poriluka) i s maslinovim uljem? _______ |

**MEDAS questionnaire** (English version [23])

| 1. | Do you use olive oil as the principal source of fat for cooking?  1) No 2) Yes |
| --- | --- |
| 2. | How much olive oil do you consume per day (including that used in frying, salads, meals eaten away from home, etc.)? __________ tablespoons |
| 3. | How many servings of vegetables do you consume per day? (A full serving is 200 g; count garnish and side servings as 1/2 servings; _______ servings, out of which I daily consume ________ serving of raw vegetables or salad |
| 4. | How many pieces of fruit (or cups in case of berries, including fresh-squeezed juice) do you consume per day? __________ |
| 5. | How many servings of red meat, hamburger, or meat products (salami, sausages, etc.) do you consume per day? (One serving is 100–150 g) __________ |
| 6. | How many servings of butter, margarine or cream do you consume per day? (One serving is 12 g) __________ |
| 7. | How many carbonated and/or sugar-sweetened beverages do you consume per day? __________ |
| 8. | How much wine do you consume per week? __________glasses |
| 9. | How many servings of pulses do you consume per week? (One serving is 150 g) __________ |
| 10. | How many servings of fish/seafood do you consume per week? (One serving is 100–150 g of fish; 4–5 pieces or 200 g of seafood) __________ |
| 11. | How many times do you consume commercial (not homemade) pastry, such as cookies or cake per week? __________ |
| 12. | How many times do you consume nuts per week? (One serving is 30 g) __________ |
| 13. | Do you prefer to eat chicken, turkey or rabbit instead of beef, pork, hamburgers, or sausages?  1) No 2) Yes |
| 14. | How many times per week do you consume boiled vegetables, pasta, rice, or other dishes with a sauce made of tomato, garlic, onion, or leeks sautéed in olive oil? __________ |
