# Supplementary material for: Mediterranean diet assessment challenges: Validation of the Croatian Version of the 14-item Mediterranean Diet Serving Score (MDSS) Questionnaire
Source: PLoS One. 2021 Mar 1;16(3):e0247269. doi: 10.1371/journal.pone.0247269 (PMC7920370; doi:10.1371/journal.pone.0247269)
Supplement: S2 Table — (DOCX) [file pone.0247269.s003.docx]

**S2 Table.** **Test-retest reliability and concurrent validity of the MDSS questionnaire when participants are classified into tertiles according to the distribution**

|  | **MDSS test**  **N (%)** | | | **MEDAS test**  **N (%)** | | | **MEDAS retest**  **N (%)** | | |
| --- | --- | --- | --- | --- | --- | --- | --- | --- | --- |
| **MDSS retest** | **1^st^ tertile** | **2^nd^ tertile** | **3^rd^ tertile** | **1^st^ tertile** | **2^nd^ tertile** | **3^rd^ tertile** | **1^st^ tertile** | **2^nd^ tertile** | **3^rd^ tertile** |
| **1^st^ tertile; N (%)** | 50 (24.0) | 25 (12.0) | 4 (1.9) | 53 (25.2) | 18 (8.6) | 9 (4.3) | 49 (23.3) | 28 (13.3) | 3 (1.4) |
| **2^nd^ tertile; N (%)** | 16 (7.7) | 33 (15.9) | 10 (4.8) | 25 (11.9) | 12 (5.7) | 23 (11.0) | 23 (11.0) | 25 (11.9) | 12 (5.7) |
| **3^rd^ tertile; N (%)** | 2 (0.9) | 20 (9.6) | 48 (23.1) | 17 (8.1) | 15 (7.1) | 38 (18.1) | 18 (8.6) | 26 (12.4) | 26 (12.4) |
| **κ (*P*)** | **0.447** (<0.001) | | | 0.222 (<0.001) | | | **0.211** (<0.001) | | |
| **MEDAS retest** | **1^st^ tertile** | **2^nd^ tertile** | **3^rd^ tertile** | **1^st^ tertile** | **2^nd^ tertile** | **3^rd^ tertile** | **1^st^ tertile** | **2^nd^ tertile** | **3^rd^ tertile** |
| **1^st^ tertile; N (%)** | 40 (19.0) | 37 (17.6) | 13 (6.2) | 74 (35.6) | 14 (6.7) | 2 (0.9) |  |  |  |
| **2^nd^ tertile; N (%)** | 27 (12.9) | 26 (12.4) | 26 (12.4) | 21 (10.1) | 29 (13.9) | 28 (13.5) |  |  |  |
| **3^rd^ tertile; N (%)** | 3 (1.4) | 15 (7.1) | 23 (11.0) | 0 (0.0) | 2 (0.9) | 38 (18.3) |  |  |  |
| **κ (*P*)** | 0.127 (0.009) | | | **0.511** (<0.001) | | | n/a | | |
| **MDSS test** | **1^st^ tertile** | **2^nd^ tertile** | **3^rd^ tertile** | **1^st^ tertile** | **2^nd^ tertile** | **3^rd^ tertile** | **1^st^ tertile** | **2^nd^ tertile** | **3^rd^ tertile** |
| **1^st^ tertile; N (%)** |  |  |  | 77 (21.4) | 26 (7.2) | 17 (4.7) |  |  |  |
| **2^nd^ tertile; N (%)** |  |  |  | 73 (20.3) | 23 (6.4) | 43 (11.9) |  |  |  |
| **3^rd^ tertile; N (%)** |  |  |  | 19 (5.3) | 25 (6.9) | 57 (15.8) |  |  |  |
| **κ (*P*)** | n/a | | | **0.162** (<0.001) | | | n/a | | |
