# Supplementary material for: Mediterranean diet assessment challenges: Validation of the Croatian Version of the 14-item Mediterranean Diet Serving Score (MDSS) Questionnaire
Source: PLoS One. 2021 Mar 1;16(3):e0247269. doi: 10.1371/journal.pone.0247269 (PMC7920370; doi:10.1371/journal.pone.0247269)
Supplement: Attachment — Submitted filename: REVIEW_PONE-D-20-26357.docx [file pone.0247269.s004.docx]

This is a cross-sectional study involving two independent samples, of University students, in order to assess psychometric properties of the Croatian version of a short Mediterranean Diet questionnaire. The issue is of interest giving the importance of investigating eating habits on a large scale with simple but reliable tools. The work is well designed and the reading is sliding, however there are some methodological concerns and questions.

Minor issue:

1) The authors cited the paper of D.B. Panagiotakos where it is explained: “We used 11 main components of the Mediterranean diet (non-refined cereals, fruits, vegetables, potatoes, legumes, [olive oil](https://www.sciencedirect.com/topics/medicine-and-dentistry/olive-oil), fish, red meat, poultry, full fat [dairy products](https://www.sciencedirect.com/topics/medicine-and-dentistry/dairy-product) and alcohol” [<https://doi.org/10.1016/j.numecd.2005.08.006>].

The MDSS investigate the consumption of dairy product without distinguish low from medium/high content of fat (i.e. yogurt from cheese). This could results in a leak of goodness in assessing adherence to MD. Did the authors consider these occurrence in choosing MDDS? Should they explain how they can assert the adherence to MD without accounting for this distinction?

2) Similar to the above. Still referring to D.B. Panagiotakos, the MDDS des not distinguish whole from refined grains. Also if refined grains are largely consumed among population, whole grains use is the reference for the adherence to MD. Should the authors have chosen another questionnaire for their validation paper?

Major issue:

1) The authors tested the reliability of MEDAS and MDSS performing the test-retest procedure, but they didn’t assess the internal consistency of the MDDS (i.e. estimated with Cronbach’s alpha). Could the author provide this part in the methodology section?
2) Could the author, also, provide the psychometric properties of the items? (inter-item association)
