# Supplementary material for: Mediterranean diet assessment challenges: Validation of the Croatian Version of the 14-item Mediterranean Diet Serving Score (MDSS) Questionnaire
Source: PLoS One. 2021 Mar 1;16(3):e0247269. doi: 10.1371/journal.pone.0247269 (PMC7920370; doi:10.1371/journal.pone.0247269)
Supplement: Attachment — Submitted filename: Re-rew_PONE-D-20-26357R1.docx [file pone.0247269.s006.docx]

I really appreciate that the authors performed a test to assess the internal consistency of the MDSS, and I thanks them, even though the Cronbach’s alpha was mentioned as an example *“(i.e. estimated with Cronbach’s alpha)*” and did not necessarily have to be adopted given the limitation of this instrument in a multidimensional scale, as the authors reported.

Despite the original paper of Monteagudo C established a cut-off, I guess if in your population, as the authors stated, it would be more effective to use the questionnaire score as continuous rather than dichotomized. In fact the authors show that the score loads for 6 factors (tab 6) therefore it is not properly correct to use the dichotomous score, but this is just a personal reflection.

Your paper is well designed and described, it is suitable for the publication in PLOS ONE.
